# Perception of the image of the nursing profession and its relationship with quality of care

**DOI:** 10.1186/s12912-022-00830-4

**Published:** 2022-03-10

**Authors:** Keren Grinberg, Yael Sela

**Affiliations:** 1 Head of Nursing Sciences Department, Faculty of Social and Community Sciences, Ruppin Academic Center, 40250 Emek Hefer, Israel; 2 Lecturer, Nursing Sciences Department, Faculty of Social and Community Sciences, Ruppin Academic Center, Emek Hefer, Israel

**Keywords:** Nursing image, Nursing care, Quality care, Nursing profession

## Abstract

**Background:**

Good quality of care (QOC) is related to high recovery rates, fewer mistakes, and better outcomes in general. The perception of the nursing profession (NP) among nurses has many social and professional implications, and it is important to understand the implications regarding their QOC. The aim of the study was to examine whether there is a relationship between the self-image of nursing and the nurses’ QOC, and whether men and women differ in their nursing image (NI).

**Methods:**

A cross-sectional study applied among nursing teams employed in various inpatient wards: An online questionnaire was distributed and included (1) Sociodemographic details; (2) Image of the nursing profession; and (3) Nurses’ perception of their nursing care quality.

**Results:**

The results show a significant positive relationship between the NI perception among nurses and their perceptions of the QOC they provide. No sex differences were found between male and female nurses.

**Conclusion:**

This study highlights the correlation between the NP’s self-image and self-reported QOC. Health policy makers should build national programs that improve the image of nursing among nursing teams, and create an empowering and positive working environment, which would most probably improve the quality of nursing care.

## Background

In recent decades, organizational and economic changes, alongside sociological and demographic changes, have been transpiring in global health systems [1, 2]. Scientific developments, multiple innovative technologies, and an inundation of medical information have increased the public’s demands and expectations of health services. New models of caring for sick people and the aging population have expanded the definition of medical care. There is, hence, a need to cover health expenditures, on one hand, and to increase the delivery and accessibility of services and availability of medical staff, on the other hand [3].

The ability to provide solutions for the ever-increasing needs of the health system, specifically those related to the upsurge of chronic morbidity, depends on a country’s economic resilience, allocations for healthcare, and the health systems’ ability to recruit workforce, facilities and technologies to cope with the rapidly increasing trials. However, the combination of the challenges of increased chronic morbidity, on one hand, and the lack of workforce that can satisfy them, on the other hand, poses a significant challenge to even the most developed countries. The gap between the financial allocations and the increasing actual needs is a challenge that is common to most health systems worldwide [4].

Recent decades have witnessed many developments in the global nursing profession, due to the increase in life expectancy, chronic diseases, reduced hospitalization time in general, medical and technological developments, and the perception of the patient as a client. These trends have resulted in developments and progress in the nurse’s role including academization, extended responsibilities, and widespread use of technologies and electronic equipment [5]. In Israel, the most common degrees for nursing are a 4-year B.A. program leading to a Registered Nurse diploma (R.N.) and a Bachelor’s degree in nursing (B.A.), which offers theoretical and practical courses in anatomy, mental health, physiology, pediatric nursing, critical thinking, etc. The nursing programs prepare specialists to provide care for different patients. The nurse has a key role in reducing health gaps, providing a wide range of treatments on the health-illness spectrum, active involvement in health education and promotion, developing treatment and assessment programs, participation in multi-professional teams, and standing at the forefront of technology with the evolvement of online services [6].

### Nursing image (NI)

The attempt to recruit nursing personnel depends to a great extent on the image of the nursing profession, and is one of the many challenges that health systems must overcome. Ever since the nineteenth century, the issue of nurses’ image has been described as problematic, and although it has changed drastically since then, it is still not high. At first, the profession was perceived as feminine and maternal, comprising activities such as protection, care, bathing, and laundry [7]. The maternal image is still partially relevant today, and nurses’ main role is related to caring [8]. But, over the years, some changes have occurred. The profession has become academic; nurses are better educated, and aspire to independent careers. In general, the NP has become slightly more prestigious [9, 10].

The perception of the NP has many implications. Among policymakers in health systems, the image affects the allocation of resources and the development of the field. Among nurses, self-image affects relationships with other professions, nurses’ work performance, violence against nurses, the public’s trust in the health system, low pay and work overload, burnout, and work satisfaction [11]. Studies have found that most active nurses have a positive self-image of their profession, and only a minority have a negative one. Most feel pride in their chosen profession, although a small percentage have negative feelings such as shame [12].

It has been shown that the decision to become a nurse, and remain in the profession over time, is significantly related to the profession’s image, from both the nurse’s and the public’s perspective [11, 13]. The image of the NP affects the health system. For example, a positive NI influences students’ choice of a nursing career [14]. A positive significant relationship was found between how nurses perceive the public NI and their own self-image [15]. Also, the perceived public image was found to affect nurses’ work performance and intentions to leave the profession. When nurses believe that their public image is positive, they work better, and display more motivation to remain in the profession [16, 17].

The question is not only about the positive or public NI, but also the perception of who is suited to a nursing career, how much expertise is needed, how hard it is, and which personality traits are required to work as a nurse. For example, nursing has always been considered a feminine profession, less suited to men. This perception impacts how the profession is perceived in general [18].

In recent years, changes have occurred, and more men are choosing a nursing career [10]. This, in turn, affects the changes in the profession’s image [19]. Men have always worked in the nursing profession, but their numbers have always been much smaller than women [20]. The number of men who choose a nursing career has been rising in the United States, England and Australia in recent years, and is expected to keep rising [19], although their numbers are still low. In Canada and the US only about 5% of the nurses are men, 10% in England, and 4% in Ireland. A relatively large number of male nurses was reported in Iran, where 23% of the nurses are men [21].

As mentioned, the small number of men in nursing affects its image. Professions with fewer men are considered ‘female’, and thus related less to power and more to caring. Men are typically managers, whereas women are typically in caring professions [11]. Studies have found differences between men and women’s assessment of the nursing profession. Men appreciate the technical aspects of nursing, whereas women are more drawn to the relationships [22]. Women value the care for the patient, including respect for the patient’s autonomy, privacy and confidentiality [23]. Women also believe in a higher status of the NP [20, 24]. Yet, it is unclear if there are sex differences in self-image of the NP and self-reported quality of provided care.

In Israel, many duties and heavy workload would reduce the NI [25, 26]. It was found that most of the public perceive nursing mainly as a therapeutic profession that includes technical skills, or as a doctor’s helper. Similarly, findings elsewhere in the world have shown that nurses are perceived primarily as those who dispense medications, and listen to doctors’ instructions without asking questions [27]. The nurse’s image is affected to a large extent by how nursing is presented in the media – articles and ads in newspapers, TV series, and films. Usually, the nurse’s media image is stereotypical, and rarely presents the reality of the profession [8].

A study conducted in Vietnam [28] found a direct association between the public image of the NP and the quality of nursing care. Nurses reported that when they felt that the public was critical of them, did not respect their work, and the patients treated them in a negative way, it impacted their ability to focus on the job, their motivation declined, they made more mistakes, and their attitude towards patients was less patient and respectful.

### Quality of care (QOC)

The concept QOC is not new, and has been discussed frequently in the nursing context. However, to this day, there is no consensus on its definition, mainly because ‘quality’ is a general and somewhat vague term [29]. Quality of medical care is, thus, defined differently by various bodies, according to their agenda [30]. The American Medical Association defined quality of healthcare as a process that generates improvement or preservation of life expectancy on an ongoing basis [31].

One approach maintains that QOC includes two elements: technical quality and human-cultural quality. The technical aspect includes the actions required to achieve the desired results. This element is relatively easy to measure by professional criteria. The second element involves the quality of the relationship formed by the health professional with the patient, the communication between them, cooperation in decision making, empathy, satisfaction, and all other aspects included in the caregiver-patient relationship. This element is harder to assess [32]. Another approach defined four components of QOC: communication, human resources, patient satisfaction, and control of infections [29, 33].

Various factors were found to predict the patients’ perceptions of QOC. The strongest predictor was the patient’s feeling that he or she was in good hands. Another important factor was the nursing team measure, which includes the intake process, a respectful and kind attitude, a feeling that the nurses listen and relate to questions and concerns, and their explanations are clear and understandable, and then, in order of importance – physical conditions and the medical staff [34]. An additional factor that contributes to the quality-of-care perception is the informal support system, particularly by family members. On the other hand, the two factors that impair the QOC are the caregivers’ fatigue due to long shifts, and burnout [35].

Most studies measure the QOC by patients’ reports, but it is no less important to examine QOC from the health professionals’ perspective. For example, Moen and colleagues (2020) found that health professionals typically report high QOC [36].

It is important to understand the factors that predict QOC, because QOC affects the treatment’s success on many parameters such as reducing mistakes, better treatment results, and better recovery percentages [37, 38]. Nurses’ QOC is essential for the success of the health system in general. Nevertheless, the health system has found it hard to improve its QOC. Various intervention programs throughout the world have produced very small improvements [37, 39]. In recent years, the Israeli healthcare system has adopted several mechanisms for promoting quality and patient safety and care at various levels. These include legislation, financial incentives, quality indicators, patient experience, prevention and control of infection, accreditation, and the widespread use of electronic medical records. In addition, outcomes are heterogeneous: quality indicators, infection control and patient experience in primary care and the emergency department have all shown substantial improvement by minimizing disparities, and achieving greater improvements in patient safety and care [40].

### Sex

In Israel, the percentage of male nurses has increased over the years. In 2009, 9% of all those licensed to engage in nursing were men, and it rose to 15% by the end of 2020 [41]. Men and women experience different difficulties and challenges in the nursing profession, the public’s attitude to them, and their support, so that their attitude towards the profession and the image they develop of it are also different [42]. Nursing has traditionally been considered a female profession, and its perception is linked to this image. Research conducted in Turkey found big differences between male and female nursing students concerning the profession’s image. Women believed that men joining the NP would not affect its image, whereas men tended to believe that men joining the NP would impact its public image negatively [43]. Also, a majority of men believed that the NP should remain female.

The QOC of nurses is central to the success of the healthcare system. Good quality treatment is associated with higher recovery rates, smaller number of errors, and better overall treatment outcomes [44]. A better understanding of the factors that affect QOC, should make the health system and its services better and more efficient. Hence, the aim of this study was to examine whether there is a relationship between the self-image of nursing and the nurses’ QOC, and whether men and women differ in their NI. Based on the literature, the following hypotheses were formed:

**H1**: A positive relationship will be found between nurses’ positive image of the NP and their QOC, so that the better the NI is, the better the QOC is.

**H2**: Differences will be found between men’s and women’s NI, so that women believe more than men in a positive image.

## Methods

This cross-sectional study was applied among 122 male and female nurses employed in various inpatient wards.

### Tools

This study utilized a three-part questionnaire, as follows: 1. Sociodemographic details; 2. Image of the nursing profession; and 3. Nurses’ perception of their nursing care quality.

#### Sociodemographic questionnaire

Respondents provided data on age, sex, marital status, religion, education, employment, and income.

#### Nursing image questionnaire

This questionnaire was based on Čukljek et al. (2017) [45], (originally created by Toth and associates (1998) [46]), and was translated into Hebrew for the purpose of this research. The questionnaire was translated into Hebrew and back-translated into English by two independent translators, in order to ensure the accuracy of the translation. Its latest version was found to be identical to the original version. In order to increase the nurses’ responses to fill out the general questionnaire, the present study used the shortened version of the questionnaire with 15 specific items (taken from the original version), which were used to examine the nursing image (Cronbach’s α = 0.861) on a 5-point Likert scale (1 = *do not agree at all* to 5 = *agree very much*),. Scores ranged from 15 to 75. Content validity was supported by a panel of experts (nurses who are researching the image of nursing), who reviewed the NIQ twice to determine the representativeness and relevance of the items. The questionnaire included items such as “The nursing profession is highly regarded by the public”, “The role of the nurses is as important as the role of the physicians”, “Nurses protect patients in the health care system”, “Nurses are always learning and developing in their profession”. Cronbach’s α of the questionnaire ranged from 0.82 to 0.89, and it was found to be valid. A high score on the questionnaire indicates a more positive NI, and a low score indicates a negative image.

#### Nurses’ perception of their nursing care quality questionnaire

This questionnaire was constructed for the purpose of the study on the basis of the literature review, and was first administered as a pilot among 15 nurses. Corrections were made as per their recommendations. Cronbach’s α of the questionnaire ranged from 0.74 to 0.86. The questionnaire was reviewed by eight content experts, and was found to be valid. The experts judged, as a panel using the Delphi technique, whether the questionnaire items adequately measured the structure to be evaluated, and whether the items were sufficient to measure the perception of the quality of care, and consensus was gained. The questionnaire examines the QOC from the nurse’s perspective, and includes 19 items on a 4-point Likert scale (1 = *very rarely* to 4 = *very often*) that address a variety of nursing activities, including nurses’ attention, kindness, respect, skills, ability, and realization of the patient’s needs. A high score on the questionnaire indicates a positive perception of nursing care quality, and a low score indicates a negative perception of nursing care quality.

### Procedure

The study was approved by the Ethics Committee of Ruppin Academic Center. All subjects signed a written informed consent form to participate in the study, after receiving an explanation of its purpose. Next, an online questionnaire constructed on the Google Forms platform was distributed on social media and nurses’ internet forums utilizing snowball sampling. One hundred twenty-two valid questionnaires were received.

### Data analysis

The data were analyzed with SPSS ver. 23. For the first hypothesis, Pearson correlation coefficients were calculated to measure of the strength of the association between the NI and the QOC, both continuous variables. For the second hypothesis, t-tests for independent samples were performed to examine the differences between men’s and women’s NI and QOC.

## Results

The 122 respondents’ age was between 20 and 69 (M = 37.4; SD = 10.66). The distribution of other sociodemographic details is presented in Table 1. Table 2 depicts the distribution of where the nurses were employed. It is clear that the participants work in a wide variety of departments and wards.

**Table 1:** Demographic data of study participants

Variable	Options	N	%
Sex	Men Women	47 75	38.5 61.5
Marital status	Single Married Divorced Widowed	17 88 11 5	13.9 72.1 9 4.1
Religion	Jewish Muslim Christian Druze	35 75 9 3	28.7 61.5 7.4 2.5
Education	First degree Second degree Third degree	92 28 2	75.4 23 1.6
Income	Below average Average Above average	20 64 38	16.4 52.5 31.1

**Table 2:** Distribution of participants’ departments or wards

Department	N	% of total
Maternity ward	12	9.84%
Internal medicine	12	9.84%
Intensive care: general, corona, cardiac, children	11	9.02%
Other / unknown	11	9.02%
Orthopedics	7	5.74%
Dialysis	7	5.74%
Emergency medicine	7	5.74%
Complex nursing	7	5.74%
Psychiatric	7	5.74%
Geriatrics / Old age facility	6	4.92%
Children	5	4.10%
Surgical	5	4.10%
Community	5	4.10%
Gynecology	4	3.28%
Urology	3	2.46%
Neurology	3	2.46%
Cardiology	3	2.46%
Recovery	2	1.64%
Operating room	2	1.64%
Oncology	1	0.82%
Public health / Family health center	1	0.82%
Delivery room	1	0.82%

Table 3 describes the results of the NI and QOC scales. The mean score of image-of-nursing was found to be relatively positive (M = 3.81), with 2.29 as the lowest score, and the highest score 4.71 out of 5. QOC was perceived as slightly lower, but also relatively high (M = 3.55).

**Table 3:** Scores of nursing image (NI) and quality of care (QOC) variables

Variable	Minimum	Maximum	Mean	SD
Nursing image (NI)	2.29	4.71	3.81	0.53
Quality of care (QOC)	2.5	4.05	3.55	0.30

The first hypothesis assumed that the better the NI was, the better the QOC would be. Associations with some demographic variables were also examined. We found a correlation between QOC and the NI (*r* = 0.578, *p* < 0.01), so that the more positive the perception of the NI was, the better the nurses’ perception of the QOC they provided was.

**Fig. 1:**
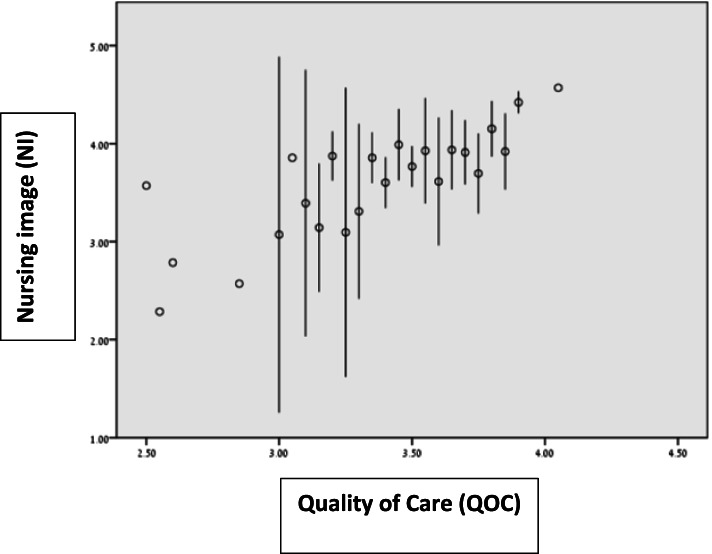
Distribution of the relationship between Nursing image (NI) and quality of care (QOC)**.** A clear linear relationship was found between NI and QOC. The more the nurses believe in a positive NI, the better the quality of their care is

We evidently found a strong, significant and positive relationship between the NI and QOC. The more the nurses believed in a positive NI, the better their quality of their care was. The first hypothesis was corroborated. The clear linear relationship between the two variables is depicted in Fig. 1.

The second research hypothesis was that differences would be found between men’s and women’s NI, so that women believed more than men in a positive image. Differences in the QOC of men and women were also examined.

Independent T-tests showed that there were no significant differences between men and women on the NI and QOC scales; therefore, the second hypothesis was not corroborated (*p* > 0.05).

Next, differences between men and women in the relationship between NI and QOC were examined. Pearson correlation coefficients were calculated for men and women separately. There was also a separate dichotomist division between men and women, and a correlation was found between the perception of the QOC and the perception of the NI among women (*r* = 0.585, *p* < 0.01) and among men (*r* = 0.587, *p* < 0.01).

## Discussion

The aim of the present study was to examine the relationship between the NI from the nurses’ perspective and the QOC they provide their patients. An additional aim was to examine whether men and women nurses differed in their self-image of nursing. These goals are important to better understand the factors that affect QOC, and can make the health system and its services better and more efficient.

The statistical analysis revealed that the nurses’ NI is relatively very high. Other studies in other countries found different results; in some, the image was revealed as positive, and in some – negative. In Jordan, for example, a positive high NI was found [15], whereas in Vietnam, the NI was negative, and had a lower status in public opinion than other professions [28]. Additionally, it was found that nurses’ self-image of their profession was more positive than the public image as they perceived it [17]. It should be noted that this is what the nurses believe the public thinks rather than a direct examination of the public’s perspective. Among the general population in Israel, nursing has higher prestige in comparison to other professions, and people of various religions perceive the profession’s prestige differently [27]. Druze and Muslims perceive the NP as more prestigious than Christians and Jews do. It is possible that the relatively high self-image found in the current study is related to the fact that 61.5% of the respondents were Muslim.

The first hypothesis was that a positive relationship would be found between nurses’ positive image of the NP and their QOC. This hypothesis was substantiated. A strong, positive relationship was found between the NI and the QOC, so that the more positive the nurse’s self-image, the better his or her QOC was. This finding supports the findings of previous studies. Takase and colleagues (2006) [17] found that nurses’ self-image was related to their work performance, so that the more positive the self-image was, his or her work behavior achieved better results. However, they did not find a relationship between how the nurses thought they were perceived by the public and their QOC. Another study in Vietnam found that a low NI had a negative effect on the nurses’ mental state – they felt more exhausted, their burnout was higher, because of a lack of public support and appreciation, and their functioning at work was compromised; i.e., the profession’s image affected the nurses’ QOC [28].

It may be that, also in Israel, the association between the NI and the QOC exists because of the perceived image’s influence on the nurses. When their work is appreciated, receives public support, and its image is positive – it boosts nurses’ resilience, they suffer less burnout, and can do their job more effectively. A positive public image contributes to nurses’ work motivation, and helps them cope with the many difficulties of the job such as overload, mistakes, complaints, and more [47]. When there is support from the organizational environment and the public, workers are willing to give more of themselves, and their work results improve considerably [48]. Hence, the current results, which indicate a direct relationship between the NI and the QOC, validate findings of other studies.

The second hypothesis assumed that a difference would be found between men’s and women’s NI, so that women perceive the NI higher than men do. This hypothesis was refuted. No differences were found for QOC either. Despite research that speaks of NI differences between men and women due to various experiences at work and vis-à-vis the public [42], in Israel no differences between men and women were found regarding the profession’s prestige [27]. These findings were corroborated in the present study. Additionally, in both groups, irrespective of sex, a strong, positive and significant relationship was revealed between NI and QOC.

Nurses are key members of healthcare teams charged to control and prevent the spread of infectious diseases, such as the COVID-19 pandemic [49, 50]. They also work on the front line, providing direct care to individuals infected with COVID-19. The multiple roles and functions played by nurses particularly during the COVID-19 pandemic may have also affected their perception of their NI, with regard to the extensive roles they performed and the authority they were given in the context of the pandemic management [51].

## Conclusion

This study indicates a clear, unequivocal relationship between nurses’ perceived NI and perceived QOC so that the better their self-image is, the better their QOC is. Nurses, who believe that their profession is perceived as positive, do a better job, engage more, listen more to patients, and in general achieve better results.

### Implications for nursing management

A long-term challenge to the NP is the concept of image. This study indicates that the image of nursing correlates with the quality of care provided by nursing staff in Israel. In-depth understanding of the NI concept would assist nurses to eliminate negative stereotypes and build a more professional image for both the individual nurse and the profession, and may affect the quality of care. However, the results of the study also have implications at a global level. Improving the nursing profession’s prestige and social position in nursing practice can change nurses’ self-image in Israel and in the world, and facilitate effective and lasting changes in nursing’s image and in the QOC nurses provide.

The image of the profession has a clear effect on the quality of the care that patients receive. As a health system that aims to provide optimal, high-quality care – it would be important to find ways to apply these conclusions in the workplace, to create an empowering and positive working environment among nursing teams, which would most probably improve the quality of nursing care as well.

### Limitations

Some limitations should be addressed. Firstly, the current sample is not representative of the Israeli population, due to the “snowball” sampling strategy and the use of a convenience sample, which excludes generalization. In addition, the sample is relatively small, and includes primarily Muslim nurses, and less Jewish, Christian or Druze nurses. Secondly, it is a cross-sectional survey, and therefore causal associations cannot be addressed. Thirdly, the respondents work in many varied departments, so it was not possible to examine the relationship between NI and QOC in a specific department. The nursing field is extensive, and it is possible that there are differences between the image and the consequent care in various departments.

## Data Availability

The datasets used and/or analyzed during the current study are available from the corresponding author on reasonable request.

## Abbreviations

NP: Nursing profession
NI: Nursing image
QOC: Quality of care
US: United States of America
SPSS: Statistical package for social science
SD: Standard deviation
